# Elucidation of reactive oxygen species scavenging pathways of norbergenin utilizing DFT approaches

**DOI:** 10.1098/rsos.221349

**Published:** 2022-12-21

**Authors:** Kautsar Ul Haq, Rahmanto Aryabraga Rusdipoetra, Imam Siswanto, Hery Suwito

**Affiliations:** ^1^ Bioinformatics Division, University CoE-Research Center for Bio-Molecule Engineering, Universitas Airlangga, Surabaya 60115, Indonesia; ^2^ Department of Chemistry, Faculty of Science and Technology, Universitas Airlangga, Indonesia

**Keywords:** scavenging mechanism, density functional theory, kinetic, antioxidant, norbergenin, Alzheimer's

## Abstract

Bergenin is a polyphenolic compound that contains isocoumarin skeletal derived from *C*-glycosylated 4-*O*-methylgallic acid. The biological activities of this compound and its derivatives are quite diverse. Recent studies reveal neuroprotective effects *in vitro* and *in vivo* in Alzheimer's. Norbergenin is a demethylated form of bergenin, known for better antioxidant capacity and associated with neuroprotective properties through oxidative stress inhibition. This study focused on investigating the scavenging mechanism of norbergenin with the ^•^OH, ^•^OOH, and $O_{2}^{∙-}$ as a radical model under physiological and lipid environments. The thermodynamic and kinetic parameters of the hydrogen transfer (HT), single electron transfer (SET), sequential proton lost-electron transfer (SPLET) and radical adduct formation (RAF) mechanisms were determined theoretically by the density functional theory (DFT) at M06-2X/6-311 + + G(d,p) level of theory. Based on the computational results, this compound has proved as an excellent ^•^OOH and ^•^OH scavenger under physiological conditions better than Trolox and vitamin C, whereas its radical demonstrated as an efficient $O_{2}^{∙-}$ scavenger.

## 1. Introduction

**Figure 1.**
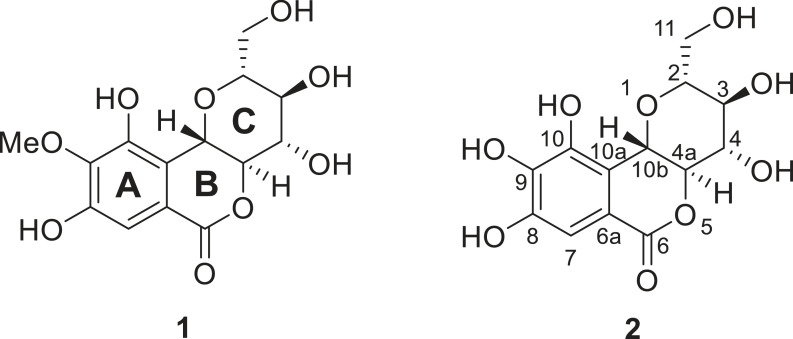
Chemical structure of bergenin (**1**), norbergenin (**2**), and structure numbering.

Bergenin (**1**) is a polyphenol compound with a tricyclic isocoumarin skeletal formed from *C*-glycosylation of 4-*O*-methylgallic acid [1]. This compound and its derivatives are commonly found in plants of the genera *Astilbe, Bergenia, Diospyros* and *Mallotus* [2]. Their biological activity is quite diverse, including antioxidant, antiviral, antifungal, antitussive, antimalarial, anti-inflammatory, antiarrhythmic, anticancer, antiulcerogenic, antidiabetic, hepatoprotective, and neuroprotective [3,4]. Norbergenin (**2**) is a demethylated form of bergenin, which exhibits better antioxidant capacity *in vitro* [5–7]. The high antioxidant capacity of **2** and its derivatives are related to neuroprotective properties through inhibiting oxidative stress via excess reactive oxygen species (ROS) neutralization [5]. Oxidative stress in neurons strongly correlates with the emergence of neurodegenerative disorders, such as Alzheimer's and Parkinson's [8,9]. Therefore, **2** and its derivatives are prospective to be developed as drug candidates to treat neurodegenerative diseases. Although it has been proven to have ROS scavenging activity *in vitro* and *in vivo*, systematic and depth theoretical mechanistic investigations of **2** as ROS scavengers have not been reported (figure 1).

Excellent antioxidant behaviour of **2** is believed to arise from pyrogallol moiety (**A** ring) [10,11]. It has been known that the phenolic group can neutralize free radicals through hydrogen transfer (HT) mechanism. The same mechanism also occurs in allylic or benzylic C–H, which plays a vital role in antioxidant properties in unsaturated compounds, such as terpenoids and unsaturated fatty acids [12–14]. The benzylic C–H in the **B** ring may be essential in antioxidant properties. The acidic pyrogallol moiety causes several parts of molecules to be deprotonated in physiological conditions. The anion formed from this process contributes to the antioxidant activity through the sequential proton lost-electron transfer (SPLET) mechanism [15]. In addition, the existence of an electron-rich aromatic system increases reactivity against ROS, which has electrophilic properties through the radical adduct formation (RAF) mechanism [16,17]. Investigation of radical scavenging activity following the mentioned mechanisms is essential to reveal the correlation between structure and antioxidant activity.

The most abundant ROS in the body is superoxide anions ($O_{2}^{∙-}$), hydroperoxyl (^•^OOH) and hydroxyl (^•^OH) radicals [18] which play an active role in oxidative stress. In the redox reactions, $O_{2}^{∙-}$ can behave both as an oxidizer (*E*°($O_{2}^{∙-}$/H_2_O_2_) = +0.93 V) and as a reducing agent (*E*°(O_2_/$O_{2}^{∙-}$) = +0.33 V) [19]. However, this anion is not very reactive and has low membrane permeability. Its conjugate acid, ^•^OOH, has a p*K*a value of 4.8 [20], and shares only 0.3% proportion under physiological conditions. Despite the small proportion, this radical has several seconds of half-lives, high membrane permeability and reacts with biomolecules at a slower rate, allowing them to diffuse into remote cell compartments [21]. By contrast, highly reactive and highly electrophilic ^•^OH radical has a short half-life (10^–9^ s) [21,22]. Therefore, this radical almost completely neutralized before diffusing to distant sites. Modelling the free radical scavenging reaction using the mentioned oxygen-centred radical models is quite a realistic approach for ROS scavenging studies.

Several theoretical antioxidant capacity studies of methylated norbergenin compounds have been reported. De Abreu *et al*. [23] studied the scavenging of several free radicals on (+)-bergenin based on a thermodynamic approach and frontier molecular orbital (FMO) theory. Ngoc *et al*. [24] conducted a more detailed theoretical investigation of 8-*O*-methylnorbergenin using the quantum mechanics for the overall free radical scavenging activity (QM-ORSA) approach. In this study, we discuss the kinetic modelling of **2** against three radicals (^•^OH, ^•^OOH and $O_{2}^{∙-}$) using the QM-ORSA approach. This study aimed to elucidate the antioxidative pathway based on structural, thermodynamic and kinetic parameters through four mechanisms (HT, SET, SPLET and RAF). In addition, the data obtained from theoretical calculations offer valuable information for performing a rational modification to improve the physico-chemical and pharmacokinetic properties without reducing the antioxidant activity.

## 2. Computational methods

All density functional theory (DFT) calculations were carried out using the Gaussian16 package [25]. The M06-2X functional was chosen because of good performance for predicting thermodynamic and kinetic parameters, especially for radical reactions [26]. Moreover, this functional has proven the most accurate for computing thermodynamic parameters in gallic acid systems [27]. The selection of flexible Pople 6-311 + + G(d,p) basis set with diffusion and polarized functions in all atoms aims to increase the calculation accuracy [28]. The unrestricted calculation was applied for open shell systems. The frequency analysis is carried out to ascertain the nature of the stationary point, where normal molecules have all real frequencies while the transition states (TSs) have only one imaginary frequency. To ensure that the found TSs are connected with two minimum points (reactants and products), the intrinsic reaction coordinate (IRC) calculations were performed. To simulate physiological conditions and lipid environments, the single-point calculations were performed at the same theoretical level in water and benzene medium with the SMD implicit solvation model as recommended by Truhlar *et al*. [29]

The intrinsic thermodynamic parameters that represent radical scavenging properties with HAT mechanism (bond dissociation enthalpy, BDE), SETPT (ionization potential, IP and proton dissociation enthalpy, PDE) and SPLET (proton affinity, PA and electron transfer enthalpy, ETE) can be determined from the enthalpy of the following reaction (equations (2.1)–(2.5)):

HAT:

2.1 $$\mathrm{BDE}=H(R^{∙})+H({\;}^{∙}H)\text{–}H(R(O)\text{–}H),$$

SETPT:

2.2 $$\;\mathrm{IP}=H(R(E)\text{–}H^{∙+})+H(e^{\text{–}})\text{–}H(R(O)\text{–}H)$$

2.3 $$\;\mathrm{and}\;\mathrm{PDE}=H(R{\left(O\right)}^{∙})+H(H^{+})\text{–}H(R(O)\text{–}H^{∙+}),$$

SPLET:

2.4 $$\;\mathrm{PA}=H(R{\left(O\right)}^{\text{–}})+H(H^{+})\text{–}H(R(O)\text{–}H)$$

2.5 $$\mathrm{and}\;\;\mathrm{ETE}\;=H(R{\left(O\right)}^{∙})+H(e^{\text{–}})\text{–}H(R{\left(O\right)}^{\text{–}}),$$

where *H*(R(O)–H), *H*(R(O)^•^), *H*(R(O)–H^•+^) and *H*(R(O)^–^) are the calculated enthalpy of the molecule, radicals, radical cations and anions, respectively. The values of *H*(H^•^), *H*(e^–^) and *H*(H^+^) in aqueous and benzene solvents were obtained from the experiment [30]. Zero-point energy (ZPE) correction were included in this calculation.

As a weak acid, this molecule can be ionized to form its anion under physiological conditions, which also has an essential role in radical scavenging activity. Theoretically, the p*K*a of **2** can be predicted by the proton exchange method using a known p*K*a reference compound with a similar structure. Propyl gallate (p*K*a = 8.11) [31] was chosen as the reference (HRef), and the prediction of the p*K*a based on the proton exchange reaction with the HRef is as follows:

2.6 $$\Delta G_{\mathrm{xc}}=[G({\mathrm{RO}}^{\text{–}})+G(\mathrm{HRef})]\text{–}[G(\mathrm{ROH})+G({\mathrm{Ref}}^{\text{–}})],$$

where *G*(RO^–^), *G*(HRef), *G*(ROH) and *G*(Ref^–^) are the calculated Gibbs energy of anion, reference, molecule and reference anion, respectively. The predicted p*K*a of **2** can be calculated from Gibbs energy of proton exchange (Δ_r_*G*_xc_) by equation (2.7).

2.7 $$pK_{a}=\frac{\Delta G_{\mathrm{xc}}}{RT\ln(10)}+pK_{a}\;\mathrm{HRef}.$$

The feasibility of a particular site to scavenge ROS can be predicted from the Gibbs free energy reaction between **2** and the radical models with the four mentioned mechanisms. For the HT mechanism, ^•^OOH was used as a radical model. In the SET and SPLET mechanism, and RAF, ^•^OH and ^•^OOH were used as models. The reactions are shown in equations (2.8)–(2.11).

2.8 $$\Delta G_{\mathrm{HAT}}^{o}=[G(R{\left(O\right)}^{∙})+G(\mathrm{HOOH})]\text{–}[G(R(O)\text{–}H)+G({\;}^{∙}\mathrm{OOH})],$$

2.9 $$\Delta G_{\mathrm{SET}}^{o}=\;=[G(R(O)\text{–}H^{∙+})+G(\mathrm{HO}{\left(O\right)}^{\text{–}})]\text{–}[G(R(O)\text{–}H)+G({\;}^{∙}O(O)H)],$$

2.10 $$\Delta G_{\mathrm{SPLET}}^{o}=[G(R{\left(O\right)}^{∙})+G(\mathrm{HO}{\left(O\right)}^{\text{–}})]\text{–}[G(R{\left(O\right)}^{\text{–}})+G({\;}^{∙}O(O)H)]$$

2.11 $$\mathrm{and}\;\Delta G_{\mathrm{RAF}}^{o}=G(R(O)(O(O)H)H^{∙})\text{–}[G(R(O)H)+G({\;}^{∙}O(O)H)].$$

The kinetics of the radical scavenging activities were calculated according to the QM-ORSA approach [32,33]. The correction to the standard state (1 M, 298.15 K) was calculated by equation (2.12). The solvent cage effect is incorporated using the correction proposed by Okuno [34] (equation (2.13)) taking into account the free volume theory proposed by Benson [35]. These two corrections decreased the free energy value by 4.55 kcal mol^−1^.

2.12 $$\Delta G_{\;}^{1M}=\Delta G_{\;}^{1\mathrm{atm}}-RT\ln(V_{m})$$

and

2.13 $$\Delta G_{\;}^{\mathrm{sol}}≅\Delta G_{\;}^{\mathrm{gas}}-RT\{\ln[n10^{\left(2n-1\right)}]-(n-1)\}.$$

The rate constant is calculated based on the transition state theory (TST) approach [36–38], according to equation (2.14),

2.14 $$k_{\mathrm{TST}}(T)=\sigma \kappa \frac{k_{B}T}{h}e^{-\Delta G^{\text{‡}}/RT},$$

where *σ* is the number of reaction symmetry, *κ* is the transmission coefficient, which is a correction to the quantum tunnelling and is calculated by the Eckart method using the Eyringpy program [39], $\Delta G^{\text{‡}}$ is the Gibbs free energy of activation, *T* is the temperature (298.15 K), *k*_B_, *h* and *R* are Boltzmann, Planck and ideal gas constants, respectively.

In the electron transfer mechanism (SET or SPLET), the values of $\Delta G^{\text{‡}}$ were calculated according to Marcus theory for electron transfer (equation (2.15)) [40].

2.15 $$\Delta G_{\mathrm{ET}}^{\text{‡}}=\;\frac{\lambda }{4}{\left(1+\frac{\Delta G_{\mathrm{ET}}^{o}}{\lambda }\right)}^{2}$$

and

2.16 $$\lambda =\Delta E_{\mathrm{ET}}-\Delta G_{\mathrm{ET}}^{o}\;.$$

The energy reorganization (*λ*) was approximated using equation (2.16), where $\Delta E_{\mathrm{ET}}$ is the vertical energy difference between the products and reactants, and $\Delta G_{\mathrm{ET}}^{o}$ is the Gibbs free energy of the ET reaction [41].

For the fast reactions taking place in solution, the rate of diffusion significantly contributes to the apparent rate constant ($k_{\mathrm{app}}$). The values of $k_{\mathrm{app}}$ were calculated by the Collins–Kimball equation (equation (2.17)) [42],

2.17 $$k_{\mathrm{app}}=\;\frac{k_{D}k_{\mathrm{TST}}}{k_{D}+k_{\mathrm{TST}}},$$

where *k*_TST_ is the reaction rate constant calculated from transition state theory, and the diffusion constant (*k*_D_) was calculated from the Smoluchowski steady state (equation (2.18)) [43]

2.18 $$k_{D}^{\;}=\;4\pi R_{\mathrm{AB}}D_{\mathrm{AB}}N_{A},$$

where *R*_AB_ is the reaction distance which has the following conditions: (i) for the SET reaction, the value is assumed to be the total radius of the reactants; (ii) for HT is equal to the distance between two atoms (donor and acceptor) involved in hydrogen transfer in the TS structure; (iii) for RAF is the distance between two atoms that will make a bond in the TS geometry [32]. *N*_A_ is Avogadro's number. *D*_AB_ is the mutual diffusion coefficient of antioxidants A and radicals B. The value of *D*_AB_ can be determined from the individual *D*_A_ and *D*_B_ as proposed by Truhlar [44], where the values of *D*_A_ and *D*_B_ are calculated by the Einstein–Stokes Equation (equation (2.19)) [45,46],

2.19 $$D_{A\;\mathrm{or}\;B}^{\;}=\;\frac{k_{B}T}{6\pi \eta r_{A\;\mathrm{or}\;B}},$$

where *η* is solvent viscosity (water = 8.91 × 10^–4^ Pa s, benzene = 6.04 × 10^–4^ Pa s) and *r*_A or B_ is solute radius of A or B.

To distinguish HAT and PCET in the hydrogen transfer process, calculations of partial charge and spin density were performed using the NPA [47], ESP [48] and Hirshfeld [49] methods were calculated in stationary points. Hirshfeld spin density and charge were also computed during IRC. The shape of the frontier molecular orbitals (SOMO and HOMO) were visualized using GaussView 6.0.16.

## 3. Results and discussions

### 3.1. Conformational analysis and acid-base equilibrium

**Figure 2.**
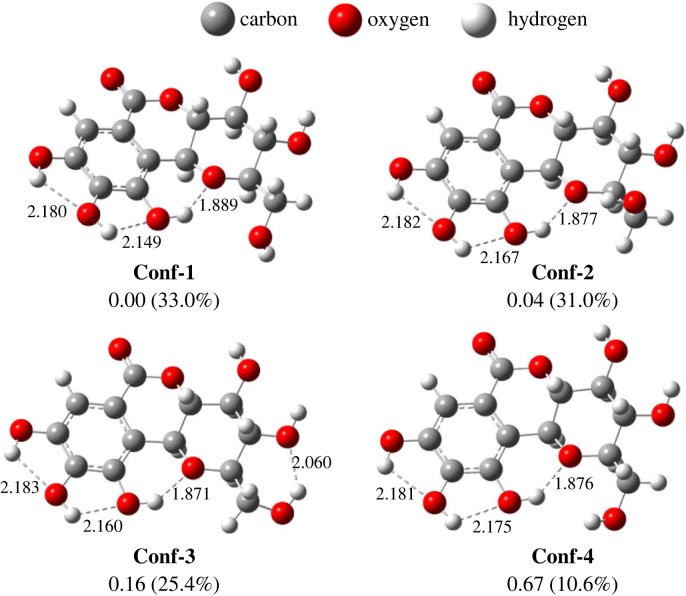
The structure of all four conformers with the relative free energy (in kcal mol^−1^) and Boltzmann distribution (in brackets). Hydrogen bonds are shown as dashed lines, and their distances are shown in Å.

Since norbergenin has several rotatable bonds, this compound can exist in several conformer structures. The systematic conformational analysis resulted in four conformers (**Conf-1** to **4**) within 2.5 kcal mol^−1^ energy from the lowest conformer. These conformers have a slight energy difference due to high structural similarity. The energy difference arises due to the high flexibility of the C_11_–C_2_ bond. All phenolic groups possess intramolecular hydrogen bonds in all conformers. Rings **A**, **B** and **C** form a planar, half-chair and chair conformation. The structure of **Conf-1** is the lowest energy conformer and is the most similar to the crystal structure [50]. Therefore, for all calculations in this study, the **Conf-1** model was used (figure 2).

As a weak acid, this compound has three deprotonable phenolic groups. Based on the PA value (table 1), the 9-OH was predicted to be the easiest to be deprotonated due to the lowest value (31.09 kcal mol^−1^). Prediction of p*K*a in this site, employing the exchange method using propyl gallate as a reference, produced a value of 8.46, not much different from the p*K*a_2_ of gallic acid, which is 8.24 [16]. At physiological pH, approximately 92% are in molecular form (H_3_A), and the remaining 8% are in monoanionic form (H_2_A^–^). The second and third deprotonations were not considered, because the similar system (gallic acid) has p*K*a_3_ and p*K*a_4_ values of 9.97 and 13.11, respectively. These values indicated that the second and third deprotonations produce HA^2–^ and A^3–^ anion in negligible molar fractions (less than 0.001).

**Table 1.** Gibbs free energy and enthalpy of HAT, SET, SPLET and RAF reactions Δ*G*_r_ and Δ*H*_r_.

site	Δ*G*_r_		Δ*H*_r_	
	water	benzene	water	benzene
HT 10b-CH	−12.28	−2.57	−6.52	3.18
HT 8-OH	−9.62	2.40	−4.57	7.44
HT 9-OH	−13.53	−3.07	−8.58	1.88
HT 10-OH	−9.74	4.38	−3.92	10.20
SET	27.44	—	32.14	—
SPLET	−2.67	—	1.93	—
RAF C-6a	18.62	26.28	11.59	19.25
RAF C-7	6.62	17.36	−0.10	10.64
RAF C-8	10.58	20.46	3.47	13.35
RAF C-9	4.31	13.24	−3.13	5.81
RAF C-10	9.91	18.68	2.68	11.45
RAF C-10a	12.20	20.84	4.20	12.85

### 3.2. Evaluational of antioxidant potential via intrinsic thermochemical parameters

Firstly, the calculation of five intrinsic parameters (BDE, IP, PDE, PA and ETE) both in aqueous and benzene solvents was conducted to find sites with high potential to scavenge free radicals. As shown in electronic supplementary material, table S1, it is determined that there are four sites with excellent scavenging ability following the HT mechanism in water (BDE is approx. 72.89–77.55 kcal mol^−1^). The order is 9-OH > 10b-CH > 8-OH > 10-OH, correlated with the stability of the formed radicals. This stability is affected by spin density distribution [51,52], as seen in electronic supplementary material, figure S2, where the 9-O^•^ and 10b-C^•^ have more distributed spin density due to resonance stabilization by the ester group.

In the four lowest BDEs, 10-OH has the highest BDE value because of the strong H-bond to O1, indicated by the shortest H-bond (1.889 Å). Therefore, extra energy is needed to break up this bond during the HT process [53]. The same trend is also observed in benzene, with consistently higher, approximately 2–6 kcal mol^−1^ (BDE approx. 75.41–83.74 kcal mol^−1^). A slight difference in BDE values between the two media indicated HT mechanism is not significantly affected by medium polarity. The results showed that the radical scavenging process by this mechanism occurs more easily in water than in a lipid environment.

To determine the preferred electron transfer mechanism, IP and PDE values were used to evaluate the SETPT, while PA and ETE values were used to assess SPLET. The results showed that the IP value is larger than PA or ETE values. Furthermore, the IP value in water and benzene are 118.12 and 162.76 kcal mol^−1^, respectively. The PA value ranges between 31.09 and 89.45 kcal mol^−1^ (water) and 83.81 and 134.21 kcal mol^−1^ (benzene), while the ETE value ranges are approximately 40.80–99.45 kcal mol^−1^ (water) and 49.86–97.66 kcal mol^−1^ (benzene). From these facts, the radical scavenging process tends to occur via the SPLET mechanism. Therefore, kinetic modelling must consider this mechanism, although the anion molar fraction is only 8%. In addition, the fact that the electron transfer process occurs more easily in polar medium should be taken in consideration, as shown by the PA and ETE values which are consistently lower in water than in benzene.

### 3.3. Feasibility study of ^•^OOH scavenging sites via thermodynamic parameters

To ensure that radical scavenging occurs in particular sites, an evaluation based on the free energy of the reaction must be carried out. Negative free energy indicates a spontaneous reaction under standard conditions. Based on Δ*G* values shown in table 1, it is known that the HT process at 8-OH, 9-OH, 10-OH, 10b-CH and SPLET takes place spontaneously and is feasible for kinetic investigation. In the benzene solvent, only two sites (9-OH and 10b-CH) are spontaneous according to this mechanism. However, we still must calculate the kinetic parameters, as suggested by Galano & Alvarez-Idaboy [32], who stated that the slightly positive value of Δ*G* (less than 10 kcal mol^−1^) distinction may have significant impact on kinetic parameters.

The significant difference in IP values for water and benzene solvents indicates that it is very difficult for the SET process to occur in a non-polar environment. At this condition, the acid-base dissociation was negligible [54], so the SPLET process would not happen. Based on these facts, the SET and SPLET processes are not feasible in the lipid environment. Likewise, if the RAF free energy showed all positive values, indicating that the radical scavenging with this mechanism was not spontaneous and the reaction would occur reversibly. It can be concluded that the RAF pathway was unfavourable.

### 3.4. Investigation of ^•^OOH scavenging activity based on kinetic study

**Figure 3.**
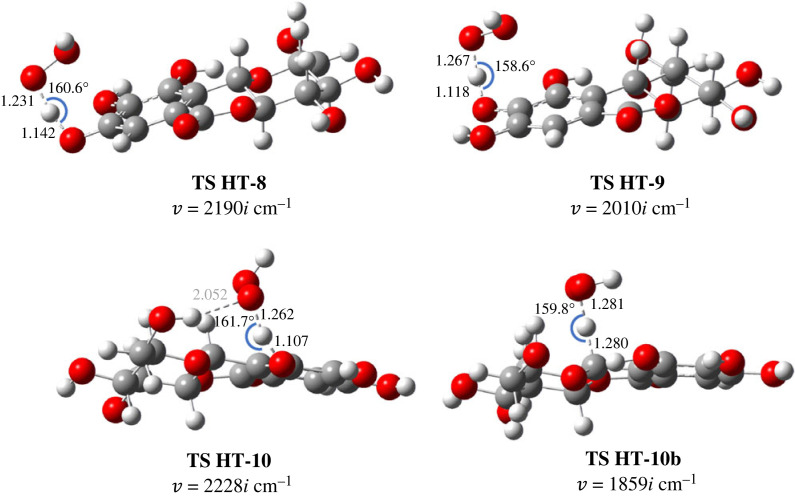
Optimized TS structures for HT reaction between **2** and •OOH in gas phase, forming and breaking bond are reported in Å and D ^…^ H ^…^ A angle is shown in degrees.

Thermodynamic studies showed that the possible mechanism in both solvents is HT, while the SPLET process can only occur in water solvents. It is well known that there is no correlation between the thermodynamic and kinetic parameters of the HT reaction. Therefore, a kinetic study is needed to quantify the contribution of 10b-CH, in which the free energy does not significantly differ from the 9-OH. The TSs structure for the HT mechanism was searched and optimized at the same theoretical level. The results of calculated kinetics for HT, SET and SPLET are shown in table 2, whereas the optimized TSs are shown in figure 3.

**Table 2.** Gibbs free energy of activation ($\Delta G_{\;}^{\text{‡}}$, kcal mol^−1^) at standard condition, calculated rate constant (*k*_TST_, M^–1^ s^–1^), diffusion rate constant ($k_{D}^{\;}$, M^–1^ s^–1^) and apparent rate constant (*k*_app_, kcal mol^−1^) in water (W) and benzene (Bz).

mechanism	$\Delta G_{\;}^{\text{‡}}$		*κ*		$k_{\mathrm{TST}}^{\;}$		$k_{D}^{\;}$		$k_{\mathrm{app}}^{\;}$	
	W	Bz	W	Bz	W	Bz	W	Bz	W	Bz
HT 8-OH	9.39	18.44	39.1	68	3.35 × 10^8^	1.36 × 10^7^	3.17 × 10^9^	3.04 × 10^9^	3.35 × 10^8^	1.36 × 10^2^
HT 9-OH	8.54	16.68	17.9	116.1	6.50 × 10^8^	4.52 × 10^3^	3.18 × 10^9^	3.04 × 10^9^	6.50 × 10^8^	4.52 × 10^3^
HT 10-OH	12.62	20.09	91.5	20.8	3.40 × 10^6^	2.59	3.17 × 10^9^	3.03 × 10^9^	3.40 × 10^6^	2.59
HT 10b-CH	11.89	19.16	34	73.5	4.33 × 10^6^	43.8	3.41 × 10^9^	3.27 × 10^9^	4.33 × 10^6^	43.8
SET	27.68	—	22.8*^a^*	—	3.39 × 10^−7^	—	8.25 × 10^9^	—	3.39 × 10^−7^	—
SPLET	0.35	—	22.1*^a^*	—	4.91 × 10^10^	—	8.24 × 10^9^	—	7.05 × 109	—

^a^reorganization energy (λ) for SET and SPLET reaction.

Although the rate constants are significantly different, the order of these values shows a consistent pattern: 9-OH > 8-OH > 10b-CH > 10-OH. Among the four sites, 10-OH is the most difficult to be abstracted by ^•^OOH. The argument is the same as its higher BDE value: the strong H-bond. Meanwhile, the H-bond in 8-OH and 9-OH are longer, 2.180 and 2.149 Å, respectively (figure 2), which explains that weaker H-bond tends to more easily donate H atom. Almost all broken bonds have shorter lengths than the formed bonds in the TS structures, indicating an early character, except on the TS HT-10b. By contrast, TS HT-10 is the earliest, but has the highest $\Delta G_{\;}^{\text{‡}}$. This case occurs because TS of HT-10 contains H-bonds between ^•^OOH and 11-OH, thus bringing this radical closer to the 10-OH.

The overall rate constants in water ($k_{\mathrm{overall}}^{W,\mathrm{pH}=7.4}$) and benzene ($k_{\mathrm{overall}}^{\mathrm{Bz}}$) were computed to determine the contribution of each mechanism. The contribution of each mechanism is expressed in branching ratio (Γ), which can be calculated by equations (3.1)–(3.4).

3.1 $$k_{\mathrm{overall}}^{W,\mathrm{pH}=7.4}=\;f_{\left(H_{3}A\right)}(\Sigma k_{\mathrm{app}}^{W,\;\mathrm{HT}}+k_{\mathrm{app}}^{W,\mathrm{SET}})+f_{\left(H_{2}A-\right)}k_{\mathrm{app}}^{W,\mathrm{SPLET}},$$

3.2 $$k_{\mathrm{overall}}^{\mathrm{Bz}}=\;\Sigma k_{\mathrm{app}}^{\mathrm{Bz},\;\mathrm{HAT}},$$

3.3 $$\Gamma_{W,\mathrm{pH}=7.4}=\frac{\;fk}{k_{\mathrm{overall}}^{W,\mathrm{pH}=7.4}}\times 100\text{\%}$$

3.4 $$\;\mathrm{and}\;\Gamma_{\mathrm{Bz}}=\frac{k}{k_{\mathrm{overall}}^{\mathrm{Bz}}}\times 100\text{\%},$$

where $f_{\left(H_{3}A\right)}$ and $f_{\left(H_{2}A-\right)}$ are the molar fraction of molecule and anion, while $\Sigma k_{\mathrm{app}}^{W,\;\mathrm{HT}}$, $k_{\mathrm{app}}^{W,\mathrm{SET}}$, $k_{\mathrm{app}}^{W,\mathrm{SPLET}}$ and $\Sigma k_{\mathrm{app}}^{\mathrm{Bz},\;\mathrm{HT}}$ are the total rate constants of HT, SET, SPLET in water and HT in benzene, respectively. The results are presented in table 3.

**Table 3.** Apparent rate constant ($k_{\mathrm{app}}^{\;}$, M^–1^ s^–1^), molar fraction (*f*), molar fraction-weighted rate constant ($k_{f}^{\;}$, M^–1^ s^–1^), overall rate constant ($k_{\mathrm{overall}}^{\;}$), and branching ratio (Γ, %) of HT, SET and SPLET mechanism in water (pH = 7.4) and benzene.

mechanism	water			benzene		
	$k_{\mathrm{app}}^{\;}$	*f*	$k_{f}^{\;}$	Γ (%)	$k_{\mathrm{app}}^{\;}$	Γ (%)
HT 8-OH	3.35 × 10^8^	0.921	3.09 × 10^8^	21.0	1.36 × 10^2^	2.9
HT 9-OH	6.50 × 10^8^	0.921	5.99 × 10^8^	40.6	4.52 × 10^3^	96.1
HT 10-OH	3.40 × 10^6^	0.921	3.13 × 10^6^	0.2	2.59	0.1
HT 10b-CH	4.33 × 10^6^	0.921	3.98 × 10^6^	0.3	43.8	0.9
SET	3.39 × 10^−7^	0.921	3.12 × 10^−7^	∼0.0	—	—
SPLET	7.05 × 10^9^	0.079	5.59 × 10^8^	45.4	—	—
*k*_overall_			**1.47 × 10^9^**		**4.71 × 10^3^**	

Based on these data, the $k_{\mathrm{overall}}^{W,\mathrm{pH}=7.4}$ is close to diffusion control, while the $k_{\mathrm{overall}}^{\mathrm{Bz}}$ is lower by six orders of magnitude (1.47 × 10^9^ versus 4.71 × 10^3^ M^–1^ s^–1^). Thus, this compound behaves as a good scavenger in the polar medium but drastically reduces when in the non-polar medium. It can be seen from the branching ratio that SPLET competes with HT (45.4 versus 54.6%) in aqueous solvent. However, in benzene, HT is the only reliable mechanism. For the HT process, 9-OH consistently has a significant contribution in both solvents, especially in benzene which contributes approximately 96.1%. Despite having excellent thermodynamic parameters, the Γ value of 10b-CH is below 1%, indicating this site has a negligible impact on antioxidative properties. Such contrasting thermodynamics and kinetics phenomenon of CH abstraction were also found in artepillin C [55], some lignans [56] and gnetin C [57].

The kinetic analysis has proven that the phenolic groups significantly contribute to the ^•^OOH scavenging activity. The modification in **A** ring strongly affects its activity. Methylation in 9-OH greatly reduced the antioxidant activity (IC_50_ = 921 versus 13 µM in DPPH assay) [5], but in 8-OH slightly reduced its activity ($k_{\mathrm{overall}}^{\;}$ for water and pentyl ethanoate approximately 8.14 × 10^8^ and 3.02 × 10^2^ M^–1^ s^–1^, respectively) [24]. Methylation at the 8-OH retains the catechol group, which still has significant antioxidant activity. The small contribution of 10b-CH has a positive effect because the number of carbon-centred radicals from radical scavenging activity is negligible. It should be noted that carbon-centred radicals tend to oxygen insertion producing reactive alkyl hydroperoxyl radicals, with a high *k* value, approximately (1–3) × 10^8^ M^–1^ s^–1^ [58]. These radicals can act as precursors for other ROS, such as alkyl hydroperoxides and alkoxyl radicals [59].

The protective effect of this molecule against ROS attack in physiological systems can be predicted by comparing the overall rate constants of biomolecules or commercial antioxidants. The rate of bisallylic hydrogen abstraction on PUFA by ^•^OOH is generally used as a threshold, where the PUFA can be considered as representative of the cell membrane. Based on the experimental results, the $k_{\mathrm{overall}}^{\;}$ value is approximately (1.18–3.05) × 10^3^ M^–1^ s^–1^ [60]. It is predicted that this molecule can neutralize ^•^OOH almost 10^6^ times faster than PUFA, so it can be said that **2** can protect cell membranes from lipid peroxidation. When compared with commercial antioxidants, this molecule is 10^5^ times more potent than Trolox ($k_{\mathrm{overall}}^{W,\mathrm{pH}=7.4}=8.96\times 10^{4}\;M^{\text{–}1}\;s^{\text{–}1}$) [61] and 10-fold stronger than vitamin C ($k_{\mathrm{overall}}^{W,\mathrm{pH}=7.4}=1.00\times 10^{8}\;M^{\text{–}1}\;s^{\text{–}1}$) [32]. The activity in the non-polar medium was slightly better than in Trolox ($k_{\mathrm{overall}}^{\mathrm{PE}}=3.40\times 10^{3}\;M^{\text{–}1}\;s^{\text{–}1}$).

### 3.5. Hydrogen transfer reaction: HAT or PCET?

**Figure 4.**
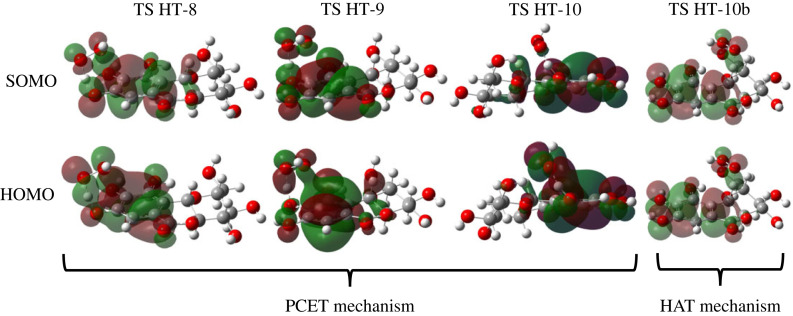
SOMO and HOMO of all four optimized TS structures in gas phase (isovalue was set at 0.02).

Generally, HAT involves a non-heteroatomic donor or acceptor; or one is a heteroatom, and in PCET both are heteroatoms [28]. Although there are evident characteristics, distinguishing the hydrogen transfer process, whether through the HAT or PCET, is not an easy task. It is advisable to perform some calculation methods [62]. The FMO approach is generally used as a preliminary evaluation. The shape of the TS singly occupied molecular orbital (SOMO) is commonly used to distinguish between the two mechanisms. In HAT, the electron densities are distributed along the transition vector of H-atom displacement, as seen in SOMO TS HT-10b (figure 4), whereas in PCET, the shape of the electron densities are orthogonal with the transition vector [63]. Unfortunately, this diagnosis is ambiguous because it is more likely to HAT in the cisoid TS structures, such as SOMO TS HT-8, 9 and 10. This case also happens for other phenolic systems calculated at the same theoretical level [28].

DiLabio & Johnson [64] recommended analysing the shape of HOMO orbital to overcome this problem. In phenolics, PCET involves electron transfer through interaction between the lone pairs of oxygen on the ^•^OOH and the aromatic π-orbital. This interaction can be seen as overlapped electron densities between lone pair ^•^OOH and aromatic π-orbital, as seen in the HOMO TS HT-8, 9 and 10. This phenomenon does not occur in HAT since the hydrogen atom transferred from the donor to the acceptor as a single particle as shown in HOMO TS-10b. The lone pairs orbital in ^•^OOH are entirely separated from the electron density of aromatic π-orbital, indicating that the abstraction of 10b-CH occurs via the HAT mechanism.

Another way to distinguish these mechanisms is using population analysis at stationary points in the reaction coordinate: reactant complex (RC), TS and product complex (PC). The partial charge and spin densities of the donor, hydrogen and acceptor atoms calculated using the three methods are shown in electronic supplementary material, table S4. An indication for the PCET involvements can be judged from the significant H atomic NPA charge on TS HT-8, 9 and 10 at 0.465, 0.475 and 0.483, respectively. Meanwhile in the TS 10b-CH, the H atom has a smaller value (0.308), which indicates the HAT mechanism [65]. For the ESP and Hirshfeld partial charges, the H atom in PCET has a value in the range of 0.316–0.358 and 0.091–0.095, respectively. In the HAT, these values are approximately 0.121 and 0.035, respectively. These results show that the H atom is transferred as a proton in the PCET mechanism.

To get clear differences between HAT and PCET, the evolution of Hirshfeld partial charge and spin density along reaction coordinate were calculated. As shown in electronic supplementary material, figure S5, the partial charges of H during the abstraction process at 8-OH, 9-OH and 10-OH were stable at approximately 0.1, while at 10b-CH, are increased from approximately 0 to 0.07. This confirms the assumption that the H atom in 8-OH, 9-OH and 10-OH is transferred as a charged particle. Then the increasing charge at 10b-CH is due to displacement of H atom from a lower electronegativity atom (C) to a higher one (O). The displacement of charged particles during the abstraction of 8-OH, 9-OH and 10-OH can be observed in the donor and acceptor charges at reaction coordinates of 0.2–0.6 bohr amu^1/2^. The charge on the donor increases and then decreases slightly. However, in the acceptor, the charge decreases and then increases slightly. This phenomenon indicates the electron moves from the donor to the acceptor as in the PCET mechanism.

Observation of the evolution of spin density along the progress of the reaction showed that all of them appeared to have the same pattern: spin density in acceptor decreases, while in donor increases, and hydrogen atom is relatively stable. The increase of the spin density in the donor is not as large as the decrease in the acceptor. This shows that the formed radical has higher stability than the ^•^OOH. The total spin density is also plotted against the progress of the reaction. The spin density during the abstraction reaction decreased drastically (0.37 at 9-OH), followed by 0.35 at 10-OH, 0.34 at 8-OH, and the smallest one is 0.19 at 10b-CH. The large decrease in the phenolic group indicates the significant contribution of H-bond with ^•^OOH, which also helps the stability of formed radicals. This H-bonding is essential in the PCET mechanism [63].

### 3.6. Investigation of ^•^OH scavenging activity based on a thermodynamic and kinetic study

Bergenin has been proven to scavenge ^•^OH actively formed from pulse radiolysis through RAF mechanism with diffusion control rate (3.33 × 10^9^ M^–1^ s^–1^ at pH 7) [66]. After the radical adduct is formed, the ^•^OH group is released as H_2_O, and the net reaction is the same as HT. Due to structural similarity, norbergenin may also assumed to have a similar scavenging mechanism. This molecule also has seven sp^2^-hybridized C atoms, which can act as RAF sites. However, the carbonyl group attack is unfavourable because the ^•^OH is the most electrophilic radical [22,67]. Thus, six remaining C atoms in **A** ring were investigated, and the results are shown in table 4.

**Table 4.** The free energy of reaction (Δ*G*) and activation ($\Delta G_{\;}^{\text{‡}}$) through RAF mechanism at standard conditions (in kcal mol^−1^), calculated rate constant ($k_{\mathrm{TST}}^{\mathrm{RAF}}$), diffusion constant ($k_{D}^{\mathrm{RAF}}$) and apparent rate constant ($k_{\mathrm{app}}^{\mathrm{RAF}}$) in M^–1^ s^–1^.

site	Δ*G*		$\Delta G_{\;}^{\text{‡}}$		*κ*		$k_{\mathrm{TST}}^{\mathrm{RAF}}$		$k_{D}^{\mathrm{RAF}}$		$k_{\mathrm{app}}^{\mathrm{RAF}}$	
	W	Bz	W	Bz	W	Bz	W	Bz	W	Bz	W	Bz
C-6a	−2.12	−2.78	7.71	8.13	1.2	1.2	1.75 × 10^8^	8.66 × 10^7^	2.28 × 10^9^	3.09 × 10^9^	1.75 × 10^8^	8.66 × 10^7^
C-7	−12.80	−10.20	4.14	7.70	1.0	1.2	6.02 × 10^10^	1.78 × 10^8^	2.35 × 10^9^	3.19 × 10^9^	2.26 × 10^9^	1.78 × 10^8^
C-8	−10.84	−9.862	6.47	7.76	1.1	1.2	1.31 × 10^9^	1.62 × 10^8^	2.31 × 10^9^	3.13 × 10^9^	1.31 × 10^9^	1.62 × 10^8^
C-9	−17.73	−17.10	3.41	4.37	1.0	1.0	2.07 × 10^11^	4.16 × 10^10^	2.44 × 10^9^	3.31 × 10^9^	2.41 × 10^9^	3.07 × 10^9^
C-10	−10.68	−10.04	5.52	6.44	1.0	1.1	5.93 × 10^9^	1.37 × 10^9^	2.34 × 10^9^	3.18 × 10^9^	1.68 × 10^9^	1.37 × 10^9^
C-10a	−7.63	−7.88	6.30	6.68	1.0	1.1	1.58 × 10^9^	9.21 × 10^8^	2.34 × 10^9^	3.18 × 10^9^	1.58 × 10^9^	9.21 × 10^8^

As shown in table 4, the OH addition reactions at all sites were spontaneous in all solvents, with the Δ*G* values ranging from −2.12 to −17.73 kcal mol^−1^ (water) and −2.78 to −17.10 kcal mol^−1^ (benzene). The Δ*G* differences between the two solvents range from 0.25–2.6 kcal mol^−1^, indicating the polarity of the medium has no significant effect. The spontaneous order between the two solvents is slightly different at C-8 and C-10, where in water it follows the following order: C-9 > C-7 > C-8 > C-10 > C-10a > C-6a and in benzene it follows the following order: C-9 > C-7 > C-10 > C-8 > C-10a > C-6a. However, these trends showed a consistent pattern, with C-9 and C-6a having the lowest and highest Δ*G* values. The same pattern is also found in gallic acid [16]. From a theoretical point of view, this reaction's exergonicity indicates the involvement of early TS, in accordance with Hammond's postulate.

**Figure 5.**
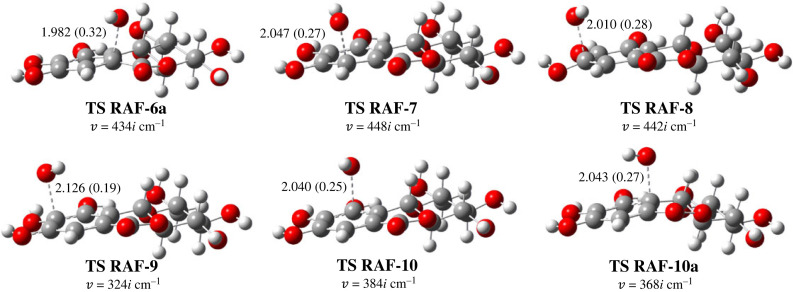
Optimized TS structures for RAF reaction between **2** and •OH radical. The forming bond is reported in Å and Wiberg bond index in parentheses.

The TSs of the RAF mechanism were determined and verified by IRC plot, as shown in electronic supplementary material, figure S3. As shown in figure 5, The Wiberg bond index for forming bonds ($R_{\mathrm{AB}}^{\mathrm{RAF}}$) are in the range of 0.19–0.32, indicating all TSs have early character. The $\Delta G_{\;}^{\text{‡}}$ in water ranged from 3.31 to 7.71 kcal mol^−1^ and higher for benzene, approximately 4.37–8.13 kcal mol^−1^. Similar to the Δ*G*, there is a difference in the order of rates between two solvents. In it water follows: C-9 > C-7 > C-8 > C-10 > C-10a > C-6a and in benzene it follows: C-9 > C-10 > C-10a > C-7 > C-8 > C-6a. From the TS structures, there is a correlation between $\Delta G_{\;}^{\text{‡}}$ and $R_{\mathrm{AB}}^{\mathrm{RAF}}$. Benzene has a better correlation (*R*^2^ = 0.88) than water (*R*^2^ = 0.79). This phenomenon can be explained based on Hammond's postulate. In this case, the TS RAF-C9 has the lowest $\Delta G_{\;}^{\text{‡}}$ due to the earliest TS, while the highest is TS RAF-C6a because of the latest TS.

As shown in table 5, the scavenging process via SPLET was preferred over SET. Although the rate is diffusion control, it has only a small contribution (8.2%) (table 6) because almost all RAF rates in water which means diffusion control (greater than 10^8^ M^−1^ s^−1^). Consistently, the position of C-9 has the largest contribution, while C-6a is the smallest one in both media. The $k_{\mathrm{overall}}^{\;}$ RAF values in the two solvents are not significantly different, so it can be concluded that **2** is active both in polar and non-polar environments. Unfortunately, it is difficult to compare the ^•^OH scavenging capacity only from the $k_{\mathrm{overall}}^{\;}$ value due to the high reactivity of this radical. Several compounds, such as tryptamine [68,69], caffeine [70] and anthranilic acid [71], behave theoretically as excellent ^•^OH scavenger. However, *in vitro* experiments proved these compounds lack antioxidant activity.

**Table 5.** The Gibbs free energy of reaction (Δ*G*) reorganization energy (*λ*) and Gibbs energy of activation ($\Delta G_{\;}^{\text{‡}}$) at the standard conditions (in kcal mol^−1^), SET rate constant $\left(k_{\mathrm{TST}}^{\mathrm{SET}}\right)$, diffusion constant $\left(k_{\mathrm{TST}}^{\mathrm{SET}}\right)$ and apparent rate constant $\left(k_{\mathrm{TST}}^{\mathrm{SET}}\right)$ in M^–1^ s^–1^.

mechanism	$\Delta G_{\;}^{\;}$	*λ*	$\Delta G_{\;}^{\text{‡},}$	$k_{\mathrm{TST}}^{\mathrm{SET}}$	$k_{D}^{\mathrm{SET}}$	$k_{\mathrm{app}}^{\mathrm{SET}}$
SET (H_3_A+^•^OH)	10.29	11.18	10.30	1.84 × 10^6^	8.72 × 10^9^	1.84 × 10^6^
SPLET (H_2_A^–^+^•^OH)	−19.82	10.50	2.07	2.00 × 10^12^	8.71 × 10^9^	8.67 × 10^9^

**Table 6.** Apparent rate constant (*k*_app_) in M^–1^ s^–1^, molar fraction (*f*), molar fraction-weighted rate constant ($k_{f}^{\;}$) in M^–1^ s^–1^ and branching ratio (Γ) in %.

mechanism	water				benzene	
	*k*_app_	*f*	*k*_f_	Γ (%)	*k*_app_	Γ (%)
RAF C-6a	1.75 × 10^8^	0.921	1.61 × 10^8^	1.7	8.66 × 10^7^	1.5
RAF C-7	2.26 × 10^9^	0.921	2.08 × 10^9^	22.2	1.78 × 10^8^	3.1
RAF C-8	1.31 × 10^9^	0.921	1.21 × 10^9^	12.9	1.62 × 10^8^	2.8
RAF C-9	2.41 × 10^9^	0.921	2.22 × 10^9^	23.7	3.07 × 10^9^	53.0
RAF C-10	1.68 × 10^9^	0.921	1.55 × 10^9^	16.6	1.37 × 10^9^	23.7
RAFC-10a	1.58 × 10^9^	0.921	1.45 × 10^9^	15.5	9.21 × 10^8^	15.9
SET	1.84 × 10^6^	0.921	1.70 × 10^6^	0.0	—	—
SPLET	2.26 × 10^9^	0.079	6.87 × 10^8^	7.3	—	—
Overall			9.36 × 10^9^		5.78 × 10^9^	

### 3.7. Investigation of $O_{2}^{∙-}$ scavenging activity based on thermodynamic and kinetic study

**Figure 6.**
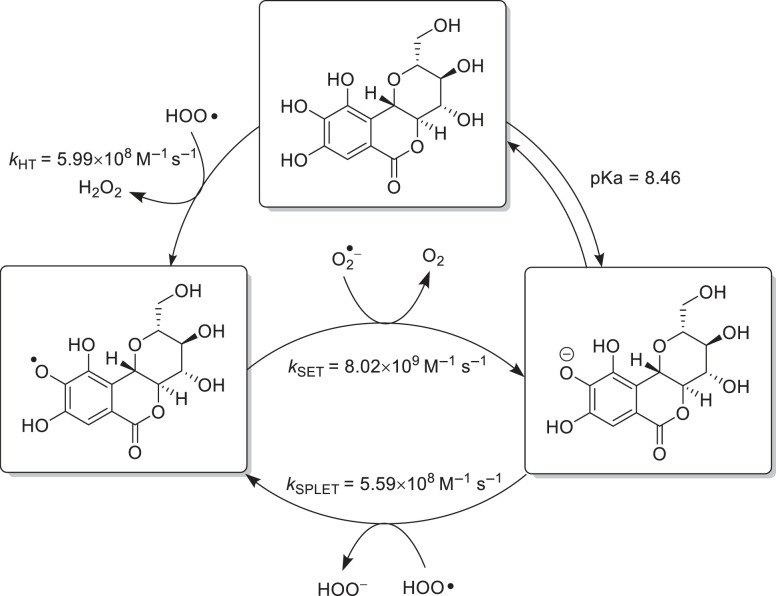
Hydroperoxyl and superoxide anion radical scavenging cycle in water medium.

Unlike the ^•^OH and ^•^OOH radicals, the $O_{2}^{∙-}$ radicals can be quenched by oxidation reaction to produce ^3^O_2_ [72]. Under physiological conditions, **2** and its anion can scavenge ^•^OOH by HT and SPLET mechanisms, producing the same product (**9-O^•^**) in diffusion control rate. This radical can behave as an oxidizer, taking electrons from other species ($O_{2}^{∙-}$), consequently **9-O**^−^ anion is regenerated. The calculation results showed $k_{\mathrm{overall}}^{\;}=5.59\times 10^{8}\;M^{\text{–}1}\;s^{\text{–}1}$. The formed anion mostly gets protonated and ready to scavenge the ^•^OOH again, forming a scavenging cycle (figure 6).

As depicted in figure 6, this molecule must react with other radicals before acting as $O_{2}^{∙-}$ scavenger. Although $O_{2}^{∙-}$ is not as reactive as ^•^OOH, it can react with H_2_O_2_ to form highly reactive ^•^OH through the Haber–Weiss reaction, albeit at a slow rate [73]. On the other hand, this anion can reduce metals, such as Cu(II) and Fe(III) to Cu(I) and Fe(II), respectively. The presence of these low valent metals can initiate the formation of ^•^OH through the Fenton reaction [74]. This reaction can produce ^•^OH faster and induce oxidative stress. The presence of **9-O^•^** will reduce the concentration of $O_{2}^{∙-}$ so that oxidative stress can be minimized.

## 4. Conclusion

The radical scavenging activity of **2** against ^•^OH, ^•^OOH and O_2_^•−^ under physiological and lipid environments was investigated by thermodynamic and kinetic calculations at the M06-2X/6-311 + + G(d,p) level of theory. From the calculation results, **2** can scavenge ^•^OOH in water and benzene with *k*_overall_ approximately 1.47 × 10^9^ and 4.71 × 10^3^ M^–1^ s^–1^, respectively. In an aqueous medium, the contribution of the SPLET mechanism competes with HT (45.4 versus 54.6%), but mainly through HT in benzene medium. The largest and smallest contributions were 9-OH and 10b-CH, respectively. The HT process in the phenolic group takes place with PCET, while HAT take place in the benzylic group. The ^•^OH scavenging through the RAF pathway was excellent in both media, whereas in water and benzene had *k*_overall_ approximately 9.36 × 10^9^ and 5.78 × 10^9^ M^–1^ s^–1^, respectively. In this mechanism, the position of C-9 also has a dominant role. The **9-O^•^** radical had an excellent superoxide scavenging activity through the SET mechanism with a diffusion control rate (*k* = 8.02 × 10^9^ M^–1^ s^–1^). It can be concluded that **2** behaves as an excellent antioxidant in physiological conditions, better than Trolox and vitamin C. For physico-chemical and pharmacokinetic improvements, we propose a chemical transformation in the **C** ring because it has negligible effect in antioxidant activity and has potential transformable functional groups.

## Data Availability

All relevant necessary data to reproduce all results in the paper are within the main text and electronic supplementary material [75].

## References

[1] Bajracharya GB. 2015 Diversity, pharmacology and synthesis of bergenin and its derivatives: potential materials for therapeutic usages. Fitoterapia **101**, 133-152. (10.1016/j.fitote.2015.01.001) 25596093

[2] Ye Y-P, Sun H-X, Pan Y-J. 2004 Bergenin monohydrate from the rhizomae of *Astilbe chinensis*. Acta Crystallogr. C **60**, o397-o398. (10.1107/S0108270104008364) 15178862

[3] Barai P, Raval N, Acharya S, Borisa A, Bhatt H, Acharya N. 2019 Neuroprotective effects of bergenin in Alzheimer's disease: investigation through molecular docking, in vitro and in vivo studies. Behav. Brain Res. **356**, 18-40. (10.1016/j.bbr.2018.08.010) 30118774

[4] Singla RK, Dhonchak K, Sodhi RK, Arockia Babu M, Madan J, Madaan R, Kumar S, Sharma R, Shen B. 2022 Bergenin ameliorates cognitive deficits and neuropathological alterations in sodium azide-induced experimental dementia. Front. Pharmacol. **13**, 994018. (10.3389/fphar.2022.994018) 36249784 PMC9556967

[5] Takahashi H, Kosaka M, Watanabe Y, Nakade K, Fukuyama Y. 2003 Synthesis and neuroprotective activity of bergenin derivatives with antioxidant activity. Bioorg. Med. Chem. **11**, 1781-1788. (10.1016/S0968-0896(02)00666-1) 12659764

[6] Zamarrud AI, Hussain H, Ahmad VU, Qaiser M, Amyn A, Mohammad FV. 2011 Two new antioxidant bergenin derivatives from the stem of *Rivea hypocrateriformis*. Fitoterapia **82**, 722-725. (10.1016/j.fitote.2011.03.002) 21406219

[7] Jouwa Tameye NS, Mvot Akak C, Mouthé Happi G, Frese M, Stammler H-G, Neumann B, Ndjakou Lenta B, Sewald N, Nkengfack AE. 2020 Antioxidant norbergenin derivatives from the leaves of *Diospyros gilletii* De Wild (Ebenaceae). Phytochem. Lett. **36**, 63-67. (10.1016/j.phytol.2020.01.012)

[8] Manoharan S, Guillemin GJ, Abiramasundari RS, Essa MM, Akbar M, Akbar MD. 2016 The role of reactive oxygen species in the pathogenesis of Alzheimer's disease, Parkinson's disease, and Huntington's disease: a mini review. Oxid. Med. Cell Longev. **2016**, 1-15. (10.1155/2016/8590578) PMC5223034 28116038

[9] Pimentel C, Batista-Nascimento L, Rodrigues-Pousada C, Menezes RA. 2012 Oxidative stress in Alzheimer's and Parkinson's diseases: insights from the yeast *Saccharomyces cerevisiae*. Oxid. Med. Cell Longev. **2012**, 1-9. (10.1155/2012/132146) PMC3371773 22701754

[10] Qiu J, Chen X, Liang P, Zhang L, Xu Y, Gong M, Qiu X, Zhang J, Xu W. 2022 Integrating approach to discover novel bergenin derivatives and phenolics with antioxidant and anti-inflammatory activities from bio-active fraction of *Syzygium brachythyrsum*. Arabian J. Chem. **15**, 103507. (10.1016/j.arabjc.2021.103507)

[11] Hsu F-L, Huang W-J, Wu T-H, Lee M-H, Chen L-C, Lu H-J, Hou W-C, Lin M-H. 2012 Evaluation of antioxidant and free radical scavenging capacities of polyphenolics from pods of *Caesalpinia pulcherrima*. Int. J. Mol. Sci. **13**, 6073-6088. (10.3390/ijms13056073) 22754350 PMC3382783

[12] Ngo TC, Dao DQ, Thong NM, Nam PC. 2016 Insight into the antioxidant properties of non-phenolic terpenoids contained in essential oils extracted from the buds of *Cleistocalyx operculatus*: a DFT study. RSC Adv. **6**, 30 824-30 834. (10.1039/C6RA02683D)

[13] Pratt DA, Tallman KA, Porter NA. 2011 Free radical oxidation of polyunsaturated lipids: new mechanistic insights and the development of peroxyl radical clocks. Acc. Chem. Res. **44**, 458-467. (10.1021/ar200024c) 21486044 PMC3124811

[14] Zheng Y-Z, Fu Z-M, Deng G, Guo R, Chen D-F. 2020 Role of C–H bond in the antioxidant activities of rooperol and its derivatives: a DFT study. Phytochemistry **178**, 112454. (10.1016/j.phytochem.2020.112454) 32692658

[15] Álvarez-Diduk R, Galano A, Tan DX, Reiter RJ. 2016 The key role of the sequential proton loss electron transfer mechanism on the free radical scavenging activity of some melatonin-related compounds. Theor. Chem. Acc. **135**, 38. (10.1007/s00214-015-1785-5)

[16] Marino T, Galano A, Russo N. 2014 Radical scavenging ability of gallic acid toward OH and OOH radicals: reaction mechanism and rate constants from the density functional theory. J. Phys. Chem. B **118**, 10 380-10 389. (10.1021/jp505589b) 25119432

[17] Lu Y, Wang A, Shi P, Zhang H, Li Z. 2015 Quantum chemical study on the antioxidation mechanism of piceatannol and isorhapontigenin toward hydroxyl and hydroperoxyl radicals. PLoS ONE **10**, e0133259. (10.1371/journal.pone.0133259) 26176778 PMC4503757

[18] Collin F. 2019 Chemical basis of reactive oxygen species reactivity and involvement in neurodegenerative diseases. Int. J. Mol. Sci. **20**, 2407. (10.3390/ijms20102407) 31096608 PMC6566277

[19] Wardman P. 1989 Reduction potentials of one-electron couples involving free radicals in aqueous solution. J. Phys. Chem. Ref. Data **18**, 1637-1755. (10.1063/1.555843)

[20] Bielski BHJ, Cabelli DE, Arudi RL, Ross AB. 1985 Reactivity of HO_2_/O^−^_2_ radicals in aqueous solution. J. Phys. Chem. Ref. Data **14**, 1041-1100. (10.1063/1.555739)

[21] Marnett LJ. 1987 Peroxyl free radicals: potential mediators of tumor initiation and promotion. Carcinogenesis **8**, 1365-1373. (10.1093/carcin/8.10.1365) 3477337

[22] Pryor WA. 1988 Why is the hydroxyl radical the only radical that commonly adds to DNA? Hypothesis: it has a rare combination of high electrophilicity, high thermochemical reactivity, and a mode of production that can occur near DNA. Free Radic. Biol. Med. **4**, 219-223. (10.1016/0891-5849(88)90043-3) 2834274

[23] de Abreu HA, Aparecida dos S, Lago I, Souza GP, Piló-Veloso D, Duarte HA, de C. Alcântara AF. 2008 Antioxidant activity of (+)-bergenin—a phytoconstituent isolated from the bark of *Sacoglottis uchi* Huber (Humireaceae). Org. Biomol. Chem. **6**, 2713. (10.1039/b804385j) 18633529

[24] Ngoc TD, Le TN, Nguyen TVA, Mechler A, Hoa NT, Nam NL, Vo QV. 2022 Mechanistic and kinetic studies of the radical scavenging activity of 5-O-methylnorbergenin: theoretical and experimental insights. J. Phys. Chem. B **126**, 702-707. (10.1021/acs.jpcb.1c09196) 35029995

[25] Frisch MJ et al. 2016 *Gaussian16 Revision C.01*. Wallingford, CT: Gaussian Inc.

[26] Galano A, Alvarez-Idaboy JR. 2014 Kinetics of radical-molecule reactions in aqueous solution: a benchmark study of the performance of density functional methods. J. Comput. Chem. **35**, 2019-2026. (10.1002/jcc.23715) 25142611

[27] de Souza GLC, Peterson KA. 2021 Benchmarking antioxidant-related properties for gallic acid through the use of DFT, MP2, CCSD, and CCSD(T) approaches. J. Phys. Chem. A **125**, 198-208. (10.1021/acs.jpca.0c09116) 33400511

[28] Amić A, Milenković D, Marković Z, Cagardová D, Rodríguez-Guerra Pedregal J, Dimitrić Marković JM. 2021 Impact of the phenolic O–H *vs.* C-ring C–H bond cleavage on the antioxidant potency of dihydrokaempferol. New J. Chem. **45**, 7977-7986. (10.1039/D1NJ00690H)

[29] Marenich AV, Cramer CJ, Truhlar DG. 2009 Universal solvation model based on solute electron density and on a continuum model of the solvent defined by the bulk dielectric constant and atomic surface tensions. J. Phys. Chem. B **113**, 6378-6396. (10.1021/jp810292n) 19366259

[30] Rimarčík J, Lukeš V, Klein E, Ilčin M. 2010 Study of the solvent effect on the enthalpies of homolytic and heterolytic N–H bond cleavage in p-phenylenediamine and tetracyano-p-phenylenediamine. J. Mol. Struct.: THEOCHEM **952**, 25-30. (10.1016/j.theochem.2010.04.002)

[31] Medina ME, Iuga C, Alvarez-Idaboy JR. 2013 Antioxidant activity of propyl gallate in aqueous and lipid media: a theoretical study. Phys. Chem. Chem. Phys. **15**, 13137. (10.1039/c3cp51644j) 23824251

[32] Galano A, Alvarez-Idaboy JR. 2013 A computational methodology for accurate predictions of rate constants in solution: application to the assessment of primary antioxidant activity. J. Comput. Chem. **34**, 2430-2445. (10.1002/jcc.23409) 23939817

[33] Galano A, Mazzone G, Alvarez-Diduk R, Marino T, Alvarez-Idaboy JR, Russo N. 2016 Food antioxidants: chemical insights at the molecular level. Annu. Rev. Food Sci. Technol. **7**, 335-352. (10.1146/annurev-food-041715-033206) 26772412

[34] Okuno Y. 1997 Theoretical investigation of the mechanism of the Baeyer-Villiger reaction in nonpolar solvents. Chemistry **3**, 212-218. (10.1002/chem.19970030208) 24022950

[35] Benson SW. 1982 The foundations of chemical kinetics. Malabar, FL: Krieger.

[36] Eyring H. 1935 The activated complex in chemical reactions. J. Chem. Phys. **3**, 107-115. (10.1063/1.1749604)

[37] Evans MG, Polanyi M. 1935 Some applications of the transition state method to the calculation of reaction velocities, especially in solution. Trans. Faraday Soc. **31**, 875. (10.1039/tf9353100875)

[38] Truhlar DG, Hase WL, Hynes JT. 1983 Current status of transition-state theory. J. Phys. Chem. **87**, 2664-2682. (10.1021/j100238a003)

[39] Dzib E, Cabellos JL, Ortíz-Chi F, Pan S, Galano A, Merino G. 2019 *Eyringpy*: a program for computing rate constants in the gas phase and in solution. Int. J. Quantum Chem. **119**, e25686. (10.1002/qua.25686)

[40] Marcus RA. 1993 Electron transfer reactions in chemistry: theory and experiment. Rev. Mod. Phys. **65**, 599-610.

[41] Galano A. 2007 Relative antioxidant efficiency of a large series of carotenoids in terms of one electron transfer reactions. J. Phys. Chem. B **111**, 12 898-12 908. (10.1021/jp074358u) 17941663

[42] Collins FC, Kimball GE. 1949 Diffusion-controlled reaction rates. J Colloid Sci. **4**, 425-437. (10.1016/0095-8522(49)90023-9)

[43] Smoluchowski MV. 1918 Versuch einer mathematischen Theorie der Koagulationskinetik kolloider Lösungen. Zeitschrift für Physikalische Chemie **92U**, 129-168. (10.1515/zpch-1918-9209)

[44] Truhlar DG. 1985 Nearly encounter-controlled reactions: the equivalence of the steady-state and diffusional viewpoints. J. Chem. Educ. **62**, 104. (10.1021/ed062p104)

[45] Einstein A. 1905 Über die von der molekularkinetischen Theorie der Wärme geforderte Bewegung von in ruhenden Flüssigkeiten suspendierten Teilchen. Ann. Phys. **322**, 549-560. (10.1002/andp.19053220806)

[46] Stokes GG. 1903 Mathematical and physical papers. Cambridge, UK: Cambridge University Press.

[47] Reed AE, Weinstock RB, Weinhold F. 1985 Natural population analysis. J. Chem. Phys. **83**, 735-746. (10.1063/1.449486)

[48] Singh UC, Kollman PA. 1983 An approach to computing electrostatic charges for molecules. J. Comput. Chem. **5**, 129-145.

[49] Marenich AV, Jerome SV, Cramer CJ, Truhlar DG. 2012 Charge model 5: an extension of Hirshfeld population analysis for the accurate description of molecular interactions in gaseous and condensed phases. J. Chem. Theory Comput. **8**, 527-541. (10.1021/ct200866d) 26596602

[50] Zamarrud AV, Anjum S, Mohammad FW, Qaiser M. 2006 Desmethylbergenin hemihydrate. Acta Crystallogr. Sect. E: Struct. Rep. Online **62**, o4626-o4628. (10.1107/S1600536806037640)

[51] Trouillas P, Marsal P, Siri D, Lazzaroni R, Duroux JL. 2006 A DFT study of the reactivity of OH groups in quercetin and taxifolin antioxidants: the specificity of the 3-OH site. Food Chem. **97**, 679-688. (10.1016/j.foodchem.2005.05.042)

[52] Belcastro M, Marino T, Russo N, Toscano M. 2006 Structural and electronic characterization of antioxidants from marine organisms. Theor. Chem. Acc. **115**, 361-369. (10.1007/s00214-006-0077-5)

[53] Benayahoum A, Amira-Guebailia H, Houache O. 2015 On the role of ethylene bridge elongation in the antioxidant activity of polyhydroxylated stilbenes: a theoretical approach. C. R. Chim. **18**, 149-159. (10.1016/j.crci.2014.04.003)

[54] Davis MM, Schuhmann PJ. 1947 Acid-base reactions in benzene and other organic solvents: behavior of bromphthalein magenta with different classes of organic bases. J. Res. Natl Bur. Stand. (1934) **39**, 221. (10.6028/jres.039.013) 20269840

[55] Boulebd H, Mechler A, Hoa NT, Nam PC, Quang DT, Vo QV. 2021 Insights into the mechanisms and kinetics of the hydroperoxyl radical scavenging activity of Artepillin C. New J. Chem. **45**, 7774-7780. (10.1039/d1nj00666e)

[56] Vo QV, Nam PC, van Bay M, Thong NM, Cuong ND, Mechler A. 2018 Density functional theory study of the role of benzylic hydrogen atoms in the antioxidant properties of lignans. Sci. Rep. **8**, 12361. (10.1038/s41598-018-30860-5) 30120382 PMC6098005

[57] Khoirunisa V, Rusydi F, Boli LSP, Saputro AG, Rachmawati H, Nakanishi H, Kasai H, Dipojono HK. 2021 Computational investigation on the ·OOH scavenging sites of Gnetin C. Food Biophys. **16**, 337-345. (10.1007/s11483-021-09666-y)

[58] Forman HJ, Davies KJA, Ursini F. 2014 How do nutritional antioxidants really work: nucleophilic tone and para-hormesis versus free radical scavenging in vivo. Free Radic. Biol. Med. **66**, 24-35. (10.1016/j.freeradbiomed.2013.05.045) 23747930 PMC3852196

[59] Zielinski ZAM, Pratt DA. 2017 Lipid peroxidation: kinetics, mechanisms, and products. J. Org. Chem. **82**, 2817-2825. (10.1021/acs.joc.7b00152) 28248497

[60] Bielski BH, Arudi RL, Sutherland MW. 1983 A study of the reactivity of HO2/O2- with unsaturated fatty acids. J. Biol. Chem. **258**, 4759-4761. (10.1016/S0021-9258(18)32488-8) 6833274

[61] Alberto ME, Russo N, Grand A, Galano A. 2013 A physicochemical examination of the free radical scavenging activity of Trolox: mechanism, kinetics and influence of the environment. Phys. Chem. Chem. Phys. **15**, 4642-4650. (10.1039/c3cp43319f) 23423333

[62] Galano A, Raúl Alvarez-Idaboy J. 2019 Computational strategies for predicting free radical scavengers' protection against oxidative stress: where are we and what might follow? Int. J. Quantum Chem. **119**, e25665. (10.1002/qua.25665)

[63] Mayer JM, Hrovat DA, Thomas JL, Borden WT. 2002 Proton-coupled electron transfer versus hydrogen atom transfer in benzyl/toluene, methoxyl/methanol, and phenoxyl/phenol self-exchange reactions. J. Am. Chem. Soc. **124**, 11 142-11 147. (10.1021/ja012732c) 12224962

[64] DiLabio GA, Johnson ER. 2007 Lone pair-π and π-π interactions play an important role in proton-coupled electron transfer reactions. J. Am. Chem. Soc. **129**, 6199-6203. (10.1021/ja068090g) 17444643

[65] Galano A, Macías-Ruvalcaba NA, Medina Campos ON, Pedraza-Chaverri J. 2010 Mechanism of the OH radical scavenging activity of nordihydroguaiaretic acid: a combined theoretical and experimental study. J. Phys. Chem. B **114**, 6625-6635. (10.1021/jp912001c) 20415502

[66] Singh U, Barik A, Priyadarsini KI. 2009 Reactions of hydroxyl radical with bergenin, a natural poly phenol studied by pulse radiolysis. Bioorg. Med. Chem. **17**, 6008-6014. (10.1016/j.bmc.2009.06.055) 19608422

[67] Lu Y, Wang W, Wang D, Bian X, Zhang H, Shi P. 2022 Reaction mechanism of ferulic acid scavenging OH and NO_2_ radicals: a theoretical study. Struct. Chem. **33**, 641-647. (10.1007/s11224-021-01855-2)

[68] Drtinova L, Dobes P, Pohanka M. 2014 Low molecular weight precursor applicable for Alzheimer disease drugs synthesis (AChE and BChE inhibition, BACE inhibition, antioxidant properties and in silico modulation). J. Appl. Biomed. **12**, 285-290. (10.1016/j.jab.2014.01.010)

[69] Bentz EN, Lobayan RM, Martínez H, Redondo P, Largo A. 2018 Intrinsic antioxidant potential of the aminoindole structure: a computational kinetics study of tryptamine. J. Phys. Chem. B **122**, 6386-6395. (10.1021/acs.jpcb.8b03807) 29775059

[70] León-Carmona JR, Galano A. 2011 Is caffeine a good scavenger of oxygenated free radicals? J. Phys. Chem. B **115**, 4538-4546. (10.1021/jp201383y) 21438616

[71] Francisco-Marquez M, Aguilar-Fernández M, Galano A. 2016 Anthranilic acid as a secondary antioxidant: implications to the inhibition of OH production and the associated oxidative stress. Comput. Theor. Chem. **1077**, 18-24. (10.1016/j.comptc.2015.09.025)

[72] Cordova-Gomez M, Galano A, Alvarez-Idaboy JR. 2013 Piceatannol, a better peroxyl radical scavenger than resveratrol. RSC Adv. **3**, 20209. (10.1039/c3ra42923g)

[73] Galano A, Reiter RJ. 2018 Melatonin and its metabolites vs oxidative stress: from individual actions to collective protection. J. Pineal Res. **65**, e12514. (10.1111/jpi.12514) 29888508

[74] Pérez-González A, Prejanò M, Russo N, Marino T, Galano A. 2020 Capsaicin, a powerful •OH-inactivating ligand. Antioxidants **9**, 1247. (10.3390/antiox9121247) 33302572 PMC7763808

[75] Haq KU, Rusdipoetra RA, Siswanto I, Suwito H. 2022 Data from: Elucidation of reactive oxygen species scavenging pathways of norbergenin utilizing DFT approaches. *Figshare*. (10.6084/m9.figshare.c.6350059) PMC9768466 36569231

